# Inactivation of glutathione *S*-transferase alpha 4 blocks *Enterococcus faecalis*-induced bystander effect by promoting macrophage ferroptosis

**DOI:** 10.1080/19490976.2025.2451090

**Published:** 2025-01-16

**Authors:** Yuanyuan Ju, Chunhua Ma, Lin Huang, Yumei Tao, Tianqi Li, Haibo Li, Mark M. Huycke, Yonghong Yang, Xingmin Wang

**Affiliations:** a Nantong Institute of Genetics and Reproductive Medicine, Affiliated Maternity and Child Healthcare Hospital of Nantong University, Nantong, Jiangsu, China; b Nantong Key Laboratory of Genetics and Reproductive Medicine, Nantong, Jiangsu, China; c Department of Gastroenterology, Affiliated Maternity and Child Healthcare Hospital of Nantong University, Nantong, Jiangsu, China; d Department of Pathology, Affiliated Maternity and Child Healthcare Hospital of Nantong University, Nantong, Jiangsu, China; e Department of Clinical Laboratory, Affiliated Maternity and Child Healthcare Hospital of Nantong University, Nantong, Jiangsu, China; f Stephenson Cancer Center, Department of Radiation Oncology, University of Oklahoma Health Sciences Center, Oklahoma City, OK, USA; g Department of Nephrology, Rheumatology, and Immunology, Nantong Children’s Hospital, Nantong, Jiangsu, China; h Department of Pediatrics, Nantong Maternity and Child Healthcare Hospital, Nantong, Jiangsu, China

**Keywords:** Glutathione *S*-transferase alpha 4, ferroptosis, *Enterococcus faecalis*, macrophage, microbiota-induced bystander effect, colorectal cancer

## Abstract

*Enterococcus faecalis*-infected macrophages produce 4-hydroxynonenal (4-HNE) that mediates microbiota-induced bystander effect (MIBE) leading to colorectal cancer (CRC). Glutathione *S*-transferase alpha 4 (Gsta4), a specific detoxifying enzyme for 4-HNE, is overexpressed in human CRC and *E. faecalis*-induced murine CRC. However, the roles of Gsta4 in *E. faecalis*-induced colitis and CRC remain unclear. Herein, we demonstrate that Gsta4 is essential for MIBE by protecting macrophages from *E. faecalis*-induced ferroptosis. *E. faecalis* OG1RFSS was used to induce colitis in *Gsta4*^−/−^ and *Il10*^−/−^*/Gsta4^−/−^* mice by orogastric gavage. Ferroptosis was assessed in Gsta4-deficient murine macrophages. We found that, unlike *Il10*^−/−^ mice, *Gsta4^−/−^* and *Il10*^−/−^/*Gsta4*^−/−^ mice colonized with *E. faecalis* failed to develop colitis or CRC. Immunofluorescent staining showed a reduction of macrophages in the lamina propria of *E. faecalis*-colonized *Il10*^−/−^/*Gsta4*^−/−^ mice, as well as decreased Gpx4 expression, indicating the occurrence of ferroptosis. Ferroptosis was further confirmed in *Gsta4*-deficient murine macrophages infected with *E. faecalis*. Moreover, Gsta4 inactivation induced the upregulation of Hmox1 and phosphorylated c-Jun while blocked Nos2 expression, leading to the accumulation of intracellular ferrous iron, lipid peroxidation and, eventually, ferroptosis. Finally, Mapk8, as a ferroptosis driver, was remarkably elevated in *E. faecalis*-infected *Gsta4*-deficient macrophages. These results suggest that Gsta4 inactivation blocks MIBE by eliminating macrophages, thereby attenuates *E. faecalis*-induced colitis and CRC.

## Introduction

Gut microbiota play an important role in the initiation of colorectal cancer (CRC).^1,2^ Several CRC-inducing pathobionts such as *Bacteroides fragilis*, *Enterococcus faecalis*, *Escherichia coli*, and *Fusobacterium nucleatum* have been extensively investigated in various animal models.^2^ Of these, *E. faecalis* is a superoxide-producing commensal that is capable of inducing colitis and CRC in interleukin (Il)-10-deficient (*Il10^−/−^*) mice through the microbiota-induced bystander effect (MIBE).^3^ When Il10 is deficient, *E. faecalis* can polarize colonic macrophages to an M1 phenotype that produces diffusible mutagens and inflammatory cytokines leading to DNA damage, mutations, chromosomal instability, and cellular transformation in bystander epithelial cells.^2–5^ In this paradigm, macrophages serve as effector cells and exert a key role in MIBE.

Colonic macrophages are composed of cells arising during embryonic development and from bone marrow-derived circulating monocytes.^6^ These cells are important components of gut mucosal immunity and help maintain gut homeostasis via interacting with gut microbiota. However, abnormal activation of colonic macrophages may dysregulate intestinal homeostasis, leading to inflammatory bowel diseases and CRC.^2^ We have shown that depletion of colonic macrophages using clodronate-containing liposomes alleviates *E. faecalis*-induced colitis and CRC.^7^ In line with these findings, the depletion of colonic macrophages in the azoxymethane/dextran sodium sulfate model also attenuated colitis and CRC,^8^ highlighting the importance of macrophages in inflammation and CRC.

Previous studies have found that *E. faecalis*-polarized macrophages produce 4-hydroxy-2-nonenal (4-HNE), a lipid peroxidation byproduct of ω-6 polyunsaturated fatty acid that helps mediate MIBE.^3,9^ Glutathione *S*-transferases alpha 4 (GSTA4) is a phase II detoxifying enzymes that specifically conjugates glutathione to 4-HNE and detoxifies this aldehyde. Single-nucleotide polymorphisms in some classes of glutathione *S*-transferase genes, including *GSTA4*, are associated with increased risk of human cancers.^10,11^ GSTA4 is highly expressed in both epithelial cells and submucosal cells during murine colitis and CRC as well as in human adenomas and invasive CRC.^12,13^ Inactivation of GSTA4 in human CRC cells increases intracellular reactive oxygen species (ROS) and susceptibility to chemotherapeutic drugs.^14^ These findings suggest that overexpression of GSTA4 protects CRC cells against damage caused by oxidative tumor microenvironment and chemotherapeutic agents. However, the impact of GSTA4 in submucosal cells, particularly in macrophages during inflammation and cancer initiation remains unclear.

Il10, an anti-inflammatory cytokine, plays a crucial role in maintaining intestinal homeostasis and regulating immune responses within the gut.^15^ Il10 protects macrophages from activation by intestinal microbiota.^16^ In inflammatory bowel disease (IBD), which includes ulcerative colitis and Crohn’s disease, dysregulation of Il10 signaling has been implicated in the exacerbation of inflammation and an increased risk of CRC.^17,18^ Il10 deficiency significantly elevates somatic gene mutations in IBD models, while increased Il10 production by macrophages can prevent colitis-associated CRC.^19,20^ However, recent studies have indicated that Il10-induced signaling may also promote the initiation of CRC and liver metastasis by inhibiting tumor suppressor genes and antitumor immunity.^21,22^ While inflammatory cytokines can induce the expression of glutathione *S*-transferases,^23^ the impact of the interaction between Gsta4 and Il10 on the development of colitis and CRC is not yet fully understood.

Given the roles of GSTA4 in detoxifying 4-HNE and preventing skin cancer, we investigated the impact of Gsta4 inactivation in *E. faecalis*-induced colitis and CRC. Our findings show that concurrent inactivation of *Gsta4* and *Il10* (*Il10*^−/−^/*Gsta4^−/−^*) in mice increases susceptibility to spontaneous colitis under specific pathogen-free conditions. However, minimal colitis is observed in either *Gsta4^−/−^* or *Il10*^−/−^/*Gsta4*^−/−^ mice following colonization with *E. faecalis*. Mechanistic studies showed that inactivation of *Gsta4* led to the inhibition of Nos2 expression, accumulation of ferrous iron and ROS, and aggravation of lipid peroxidation. This resulted in ferroptosis in *E. faecalis*-infected macrophages, thereby blocked MIBE and attenuated *E. faecalis*-induced colitis and CRC. These results reinforce the critical role of macrophages as effector cells for MIBE in the initiation of CRC.

## Materials and methods

### Cell lines and bacteria

Murine macrophage cell line RAW264.7 was purchased from the National Collection of Authenticated Cell Cultures of China (Shanghai, China). Cell cultures were maintained in DMEM medium (Gibco, Shanghai, China) supplemented with 10% fetal bovine serum (Gibco), 100 units/mL penicillin (Gibco), and 100 µg/mL streptomycin (Gibco) at 37°C under 5% CO_2_. *E. faecalis* OG1RF is a human oral isolate^24^ and OG1RFSS is a spectinomycin- and streptomycin-resistant strain derived from OG1RF that was selected on the brain heart infusion (BHI) agar plates by gradually increasing spectinomycin and streptomycin to 500 μg/ml. Both strains were cultured overnight in closed falcon tubes containing 40 ml BHI at 37°C and washed with sterile phosphate buffered saline (PBS) for subsequent experiments.

### Animal studies

Animal studies were approved by the animal care and use committees of the University of Oklahoma Health Sciences Center and Oklahoma City VA Medical Center. *Il10^−/−^* mice were purchased from the Jackson Laboratory (Bar Harbor, ME, USA). Sperm from *Gsta4^−/−^* mice were purchased from the Texas A & M Institute for Genomic Medicine (College Station, Texas, USA) and rederived by the Jackson Laboratory as heterozygous *Gsta4^+/−^* mice. Homozygous *Gsta4^−/−^* mice were created through inbreeding of heterozygous mice with deletion of *Gsta4* confirmed by PCR. The *Gsta4* and *Il10* double knockout mouse strain (*Il10^−/−^*/*Gsta4^−/−^* or DKO) was established by crossbreeding *Il10^−/−^* and *Gsta4^−/−^* mice. All mouse strains were bred on the C57BL/6J genetic background.

The occurrence of spontaneous colitis in *Il10*^−/−^/*Gsta4^−/−^* (DKO) mice was assessed based on the presence of rectal prolapse and fecal occult blood in 46 offspring across four generations. Mice exhibiting confirmed signs of colitis were euthanized for pathological evaluation within 2 weeks. Age- and sex-matched offspring from wildtype (WT), *Il10*^−/−^, and *Gsta4^−/−^* mice, all raised in the same SPF environment, were randomly selected as control groups. Colonization of *E. faecalis* was performed as previously reported.^3^ Briefly, specific pathogen-free (SPF) mice were given streptomycin (200 µg/ml) and spectinomycin (50 µg/ml) in drinking water for 1 week prior to orogastric gavage of *E. faecalis* OG1RFSS (1 × 10^9^ CFU) or sterile PBS as control. To reduce the number of mice used, we colonized *Gsta4*^−/−^ and *Il10*^−/−^/*Gsta4*^−/−^ mice, and not *Il10*^−/−^ mice, as these mice have been clearly demonstrated to develop colitis and CRC after 9-month colonization with *E. faecalis*.^3,7^ Stable colonization of *E. faecalis* OG1RFSS was maintained by streptomycin and spectinomycin in drinking water and confirmed by fecal culture. Mice were necropsied after 9 months of colonization and colons fixed with 10% formalin for staining. Pathological changes were evaluated and scored based on the number of inflammatory cells, reduction of goblets, mucosal thickness, reactive atypia/dysplasia, and cancer as previously reported.^3^

### *Fecal* E. faecalis *enumeration*

Fecal samples were collected at 1 and 9 months after *E. faecalis* colonization and suspended in 1 ml BHI containing 15% glycerol. After grinding and mixing, the fecal suspension was serially diluted with sterile PBS and 20 µl of each dilution inoculated on enterococcal agar (BD, NJ, USA) plate containing spectinomycin 100 µg/ml and streptomycin 100 µg/ml. Following incubation at 37°C, colony number of *E. faecalis* OG1RFSS was counted and colony forming units per gram of feces were calculated.

### Inactivation of Gsta4

Inactivation of Gsta4 in RAW264.7 murine macrophages was achieved using the lentiCRISPRv2 system (Addgene, MA, USA) as previously reported.^14,25^ Guide RNAs (gRNAs) targeting mouse *Gsta4* gene were designed using the CRISPRdirect online tool (https://crispr.dbcls.jp.)^26^ and cloned into the lentiCRISPRv2 vector (Supplementary Table S1). Positive transformants of *E. coli* Stbl3 competent cells were screened by PCR and confirmed by Sanger sequencing. Plasmids harboring *Gsta4*-specific gRNAs were prepared using the TaKaRa MiniBEST Plasmid Purification Kit (TaKaRa, Dalian, China). RAW264.7 cells were transfected with lentiCRISPR::gRNA636 plasmids using Lipofectamine 3000 transfection reagent (ThermoFisher Scientific, Shanghai, China) according to the manufacturer’s instructions. *Gsta4*-deficient clones were screened by adding 5 µg/ml puromycin to the culture medium and maintained with 2.5 µg/ml puromycin. Deletion of *Gsta4* was confirmed by PCR and Sanger sequencing.

### Cell treatment

RAW264.7 and *Gsta4*-deficient macrophage 2D6 cells (1 × 10^6^ cells/well) were grown in 6-well plates at 37°C overnight and then treated with *E. faecalis* OG1RF at the multiplicity of infection (MOI) of 500, or PBS as control, for 1 h. Cells were washed with sterile PBS for 5 times and cultured in a complete DMEM containing penicillin G (100 units/ml) and streptomycin (100 μg/ml) for 48 h. RNA and proteins were extracted for subsequent experiments. For ferroptosis inhibitor experiments, cells were pretreated with 1 µM ferrostatin-1 (MCE Chemical, New York City, USA) for 2 h prior to *E. faecalis* infection and maintained throughout the entire experiment. Additionally, the cells were treated with ferroptosis inducer RSL3 (MCE Chemical) at 0.5 µM for 20 h as control experiments.

### Quantitative real-time PCR

RNA was extracted using FastPure^Ⓡ^ Cell/Tissue Total RNA Isolation Kit (Vazyme, Nanjing, China) and mRNA was reversely transcribed to cDNA using HiScript III RT SuperMix kit (Vazyme) according to the manufacturer’s instructions. Real-time PCR was carried out using AceQ qPCR SYBR^Ⓡ^Green Master Mix (Vazyme) and StepOnePlus real-time PCR System (Thermo Fisher Scientific). PCR primers for qRT-PCR are listed in the Supplementary Table S1.

### Western blotting

Western blotting was conducted as previously described.^12^ In brief, whole cell proteins were extracted using cell lysis buffer containing protease inhibitors (Beyotime Biotechnology, Shanghai, China). Twenty micrograms of protein were separated by SDS-PAGE and transferred to the PVDF membrane (Merck, Beijing, China) and blocked with 5% nonfat dry milk in tris-buffered saline with 0.1% tween 20 (TBST) at room temperature for 1 h. Primary antibodies (Supplementary Table S2) were diluted with blocking buffer and incubated overnight at 4°C followed by 1-h incubation with HRP-conjugated secondary antibodies at room temperature. Signals were developed using enhanced chemiluminescence (Biosharp, Hefei, China) and captured by Odyssey Fc system (LI-COR Biosciences, Lincoln, NE, USA). The intensity of protein bands was quantified using ImageJ (National Institutes of Health, Bethesda, MD, USA).

### Immunohistochemical and immunofluorescent staining

Immunohistochemical and immunofluorescent staining was conducted and scored as previously described.^13,27^ Paraffin sections were deparaffinized and rehydrated. Epitope retrieval was carried out by boiling sections in glycine-hydrochloric acid buffer (0.1 M, pH 9.0) for 12 min and cooling down to room temperature. Sections were blocked with 5% normal goat serum (Solarbio, Beijing, China) and 5% goat anti-mouse IgG serum (Solarbio) in PBS containing 0.3% Triton^TM^ X-100 at room temperature for 1 h. Primary antibodies (Supplementary Table S2) were incubated overnight at 4°C followed by 1-h incubation with secondary antibodies. Nuclei were counterstained with Hoechst 33342. Fluorescent images were captured using a laser confocal scanning microscope (Nikon, NY, USA). For immunofluorescent (IF) staining of cells, RAW264.7 and 2D6 cells (5 × 10^4^ cells/well) were grown on the tissue culture coverslips in 24-well plates and treated as needed. Cells were fixed with 4% paraformaldehyde for 15 min, then blocked with 5% goat serum in PBS containing 0.3% Triton^TM^ X-100 at room temperature for 1 h, with subsequent staining procedures following the same protocol as described above.

### Alcian blue-periodic acid Schiff’s staining

The mucosal integrity was determined using the Alcian blue-periodic acid Schiff’s (AB-PAS) staining (Solarbio, Beijing, China). Paraffin sections were deparaffinized, rehydrated, and stained with Alcian blue for 15 min, followed by washing and treatment with periodic acid. After rinsing with tap water, sections were stained with Schiff’s reagent to visualize neutral mucins. Nuclei were then counterstained with hematoxylin, differentiated using acid alcohol, blued in Scott’s solution, and rinsed with tap water. The sections were dehydrated, cleared, and mounted for microscopy.

### Intracellular ferrous iron detection

Intracellular ferrous iron was measured using FeRhoNox-1 fluorescent staining (MKBio, Shanghai, China) according to the manufacturer’s instructions. Cells were treated with *E. faecalis* OG1RF at an MOI of 500 at 37°C for 1 h. Following three washes with HBSS, FeRhoNox-1 was added to each well at the final concentration of 5 µM and incubated at 37°C for 1 h. Fluorescent images were captured using the Leica DMi8 inverted fluorescence microscope.

### ROS detection

ROS levels were assessed using a ROS Assay Kit (Beyotime Biotechnology). Following treatment, 2’,7’-dichlorodihydrofluorescein diacetate (DCFH-DA) was added to the cells at the final concentration of 10 µM and incubated at 37°C for 30 min. Cells were washed three times with a serum-free cell culture medium to remove unbound DCFH-DA and then re-seeded into a 96-well plate at a density of 4 × 10^4^ cells/well. Fluorescence intensity was measured at 525 nm using an excitation wavelength of 488 nm on the Varioskan LUX microplate reader (ThermoFisher Scientific).

### Malondialdehyde analysis

Whole-cell lysates were prepared using cell lysing buffer (Beyotime Biotechnology) and protein concentrations were determined using a BCA protein assay kit (Beyotime Biotechnology). The relative malondialdehyde (MDA) concentration in cell lysates was determined using a Lipid Peroxidation MDA Assay Kit (Beyotime Biotechnology) in accordance with the manufacturer’s instructions. Briefly, 200 µl thiobarbituric acid working solution was mixed with 100 µl of cell lysates and boiled for 15 min. The mixture was centrifuged at 1,000 ×g for 10 min, and 200 µl of supernatant was added to a 96-well plate. Absorbance was measured at 532 nm on the Varioskan LUX microplate reader and MDA concentration in the cell lysates was calculated using an MDA standard curve.

### Bioinformatic analysis

Protein–protein interaction network was analyzed using STRING, an online protein interaction analysis tool.^28^ The network was visualized with an interaction score >0.4 based on the median confidence. In addition, macrophage-specific sub-expression of ferroptosis markers in human colorectal cancer was analyzed using Gene Expression Profiling Interactive Analysis (GEPIA2021) and the EPIC deconvolution tool.^29,30^ Furthermore, the expression of GPX4 and GSTA4 in IBD patients was analyzed using the IBD Transcriptome and Metatranscriptome Meta-Analysis (IBD TaMMA) platform.^31^

### Statistical analysis

The statistical analyses were performed using GraphPad Prism 9 software (GraphPad Software, CA, USA). All data were shown as means ± SEM from at least three independent experiments. Student’s t-test was used for comparisons between experiment and control. One-way or two-way ANOVA analysis was used for multiple factor analysis. *p* value < 0.05 was considered statistically significant.

## Results

### Il10^−/−^/Gsta4^−/−^ mice spontaneously develop colitis

**Figure 1.**
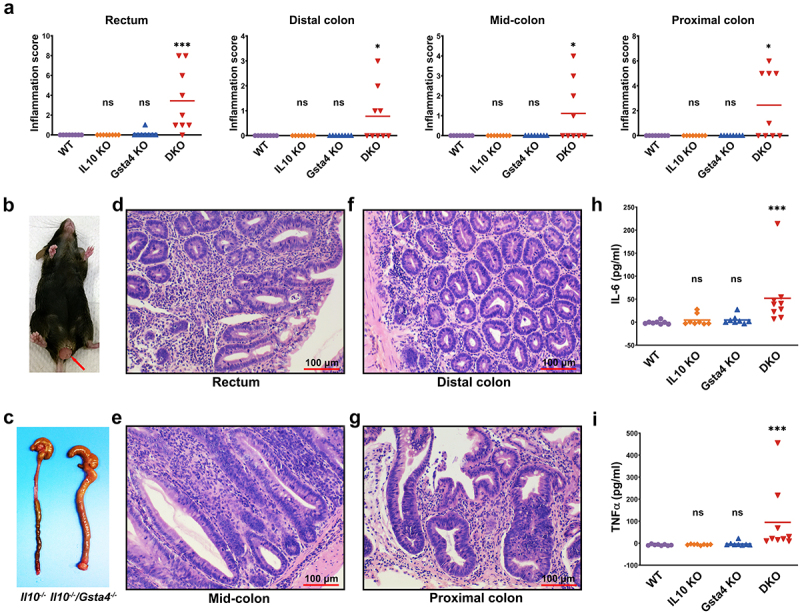
*Il10*^−/−^/*Gsta4^−/−^* DKO mice are susceptible to spontaneous colitis. (a) Colorectal inflammation scores for mice housed in SPF environment without treatment. WT, wildtype (*n* = 8); *Il10* KO, *Il10*^−/−^ (*n* = 8); *Gsta4* KO, *Gsta4*^−/−^ (*n* = 8); DKO, *Il10*^−/−^/*Gsta4*^−/−^ (*n* = 9). (b) Representative photograph of rectal prolapse in a DKO mouse. (c) Inflamed colon for *Il10*^−/−^/*Gsta4*^−/−^ (*right*) compared to normal colon for *Il10*^−/−^ mice (*left*). (d-g) H&E staining of colorectal biopsies shows severe pan-colitis. (h and i) serum Il6 and TNFα are significantly increased for DKO mice compared to control groups. *p < 0.05; ***p < 0.001; ns, not significant. All *p* values are compared to WT.

We have previously shown that *E. faecalis*-polarized macrophages produce 4-HNE that induces colitis and CRC in *Il10*^−/−^ mice via MIBE.^3,7^ Because GSTA4 specifically detoxifies 4-HNE, we explored the role of it in murine colitis-associated CRC using an *Il10^−/−^/Gsta4^−/−^* (DKO) model. Notably, neither *Il10*^−/−^ nor *Gsta4^−/−^* mice developed colitis when housed in an SPF environment (Figure 1a and Supplementary Figure S1). In contrast, 9 of 46 (19.6%) similarly housed DKO mice spontaneously developed severe rectal prolapse, a feature associated with colitis,^32^ as early as 3 months post-natal (Figure 1b). Necropsy showed inflamed colons (Figure 1c). Histopathological evaluation indicated pan-colitis in DKO mice, with dysplasia observed in only a single mouse (Figure 1a,d-g). Moreover, serum levels of TNFα and Il6 were significantly elevated in DKO mice compared to controls (Figure 1h,i). These findings indicate that the simultaneous inactivation of both *Gsta4* and *Il10* in SPF-housed mice induces spontaneous colitis.

### E. faecalis *fails to induce CRC in Il10^−/−^/Gsta4^−/−^ mice*

**Figure 2.**
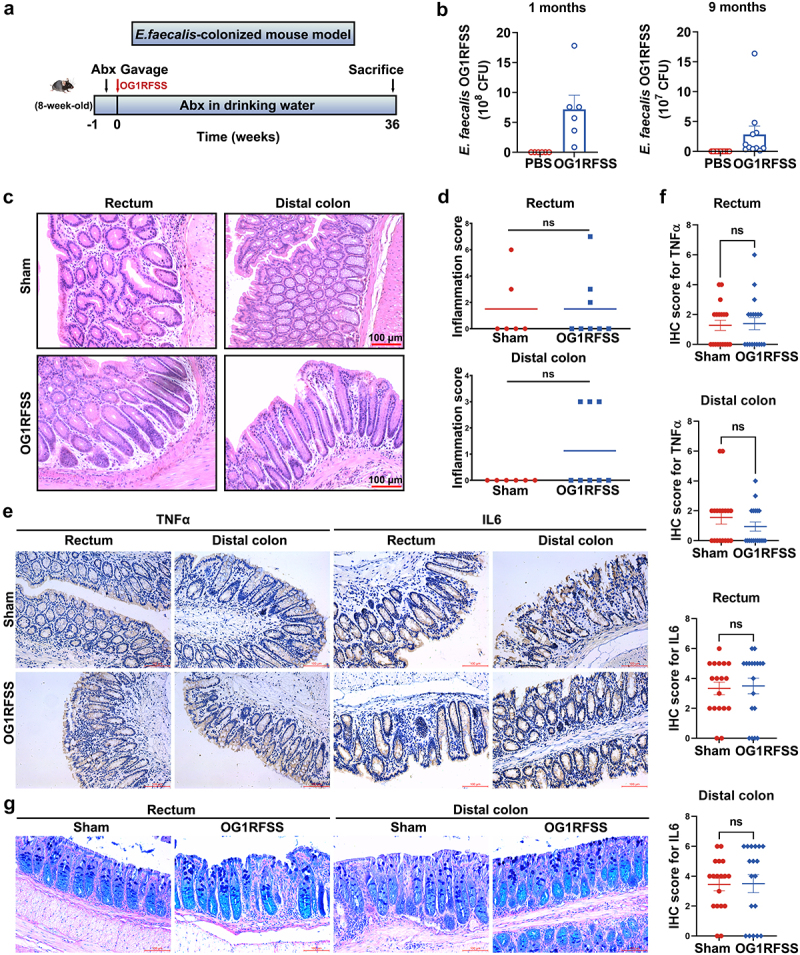
*E. faecalis* fails to induce CRC in *Il10*^−/−^/*Gsta4^−/−^* mice. (a) Schematic diagram for *E. faecalis* colonization of mice; once initiated, antibiotic-containing water was provided throughout the protocol. (b) Fecal colony counts of *E. faecalis* after 1 (*left*) and 9 months (*right*) of OG1RFSS colonization. (c) For DKO mice, H&E staining shows no evidence for colitis in colorectal biopsies from *E. faecalis* OG1RFSS- or sham-colonized mice. (d) Inflammation scores of *E. faecalis* OG1RFSS- (*n* = 8) and sham-colonized (*n* = 6) mice. (e) Representative photomicrographs of immunohistochemical staining for inflammatory cytokines TNFα and Il6 in the rectum and distal colons. (f) No significant differences are observed in the immunohistochemical staining scores between the OG1RFSS- and sham-colonized *Il10*^−/−^/*Gsta4^−/−^* mice. (g) AB-PAS staining shows mucosal integrity in colorectal biopsies from both OG1RFSS- and sham-colonized mice. ns, not significant.

Previous studies demonstrated that *E. faecalis* induces CRC in *Il10^−/−^* mice via MIBE.^3,7^ We hypothesized that inactivation of *Gsta4* would amplify oxidative stress in the colon and thereby facilitate *E. faecalis*-induced carcinogenesis in *Il10^−/−^* mice. To test this hypothesis, we colonized *Gsta4^−/−^* and *Il10^−/−^*/*Gsta4^−/−^* mice with *E. faecalis* OG1RFSS, or PBS as control, for 9 months and evaluated for colitis and CRC (Figure 2a). Mice administered *E. faecalis* maintained intestinal colonization for the duration of the experiment. Fecal colony counts showed *E. faecalis* counts at 7.18 × 10^8^ ± 5.28 × 10^8^ and 2.82 × 10^7^ ± 4.52 × 10^7^ CFU/gram stool after 1 and 9 months of colonization, respectively (Figure 2b).

While *E. faecalis*-colonized *Il10^−/−^* mice developed colitis and CRC,^3,7^ surprisingly, no increased colonic inflammation was observed in *E. faecalis*-colonized *Il10^−/−^*/*Gsta4^−/−^* mice compared to sham-treated DKO mice (Figure 2c,d) and Supplementary Figure S2). Both groups, however, tended toward decreased inflammation compared to DKO mice that were not given antibiotics as shown in Figure 1a. Immunohistochemical staining revealed minimal expression of inflammatory cytokines TNFα and Il6 in colorectal biopsies from both *E. faecalis* OG1RFSS- and sham-colonized *Il10*^−/−^/*Gsta4^−/−^* mice (Figure 2e,f). Additionally, there was no damaged mucosa or observed decrease in goblet cell numbers in the colorectal biopsies from these DKO mice following *E. faecalis* colonization (Figure 2g).

As anticipated, *Gsta4^−/−^* mice exhibited no signs of inflammation (Supplementary Figure S3A and B), with minimal expression of inflammatory cytokines TNFα and Il6 in colorectal biopsies from both *E. faecalis* OG1RFSS- and sham-treated *Gsta4^−/−^* mice (Supplementary Figure S3C-F). Similarly, no mucosal damage or decrease in goblet cell numbers was observed in *Gsta4^−/−^* mice following *E. faecalis* colonization (Supplementary Figure S3G). Finally, there was no increase in the staining of proliferating cell nuclear antigen (Pcna) in *E. faecalis*-colonized *Il10*^−/−^/*Gsta4^−/−^* and *Gsta4^−/−^* mice compared to sham-colonized controls (Supplementary Figure S4), further supporting the absence of inflammation in these mice following *E. faecalis*-colonization. These results suggest that inactivation of Gsta4 may block *E. faecalis*-induced bystander effect in *Il10^−/−^* mice, thereby attenuating inflammation and potentially preventing CRC.

### E. faecalis *induces macrophage ferroptosis in Il10^−/−^/Gsta4^−/−^ mice*

**Figure 3.**
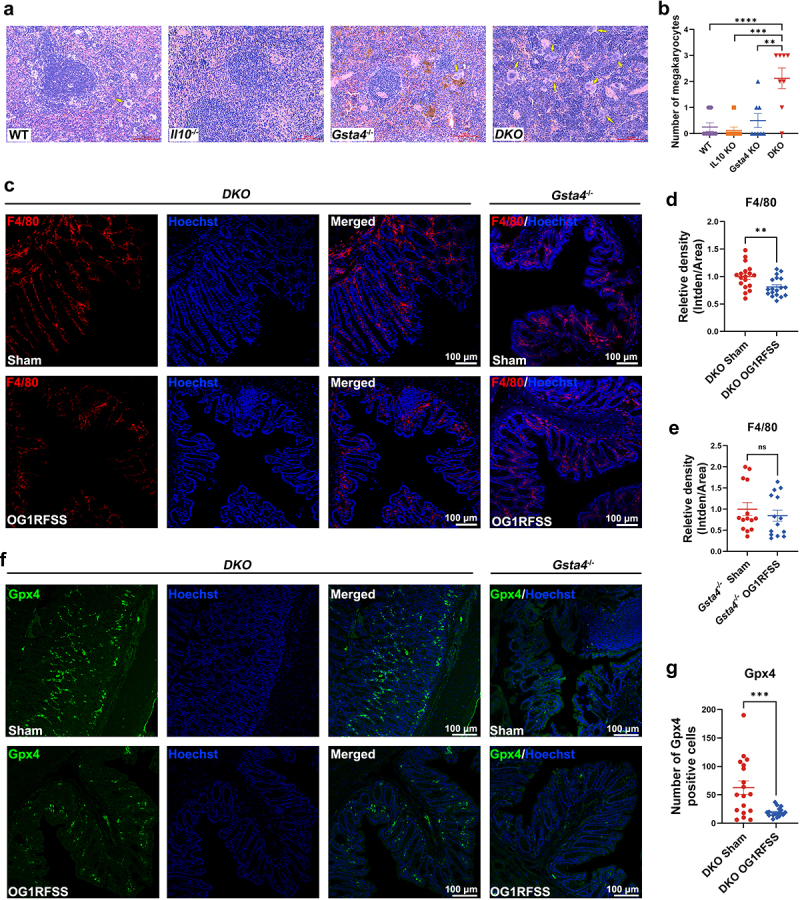
*E. faecalis* induces macrophage ferroptosis in *Il10*^−/−^/*Gsta4^−/−^* mice. (a and b) H&E staining for splenic biopsies shows increased siderophages (*brown*) in *Gsta4*^−/−^ mice and megakaryocytes (*yellow arrows*) in *Il10*^−/−^/*Gsta4^−/−^* mice. (c-e) if staining shows reduced F4/80-positive macrophages (*red*) in colon biopsies from *Il10*^−/−^/*Gsta4^−/−^* mice, but not *Gsta4^−/−^*mice, colonized with *E. faecalis* OG1RFSS compared to sham. Fluorescent intensity is analyzed in at least 3 microscope fields (20×) of 6 mice per group. (f and g) Gpx4-positive cells (*green*) are decreased in colon biopsies from *E. faecalis*-colonized DKO mice compared to sham-colonized mice, however, rare Gpx4-positive cells are seen in *Gsta4*^−/−^ mice colonized with either *E. faecalis* OG1RFSS or PBS sham. Nuclei are counterstained by Hoechst 33342. **p < 0.01 and ***p < 0.001, ****p < 0.0001; ns, not significant.

Pathological analysis of spleens from *Gsta4*^−/−^ mice found increased deposition of hemosiderin (*i.e*., siderophages) compared to WT, *Il10*^−/−^, and DKO mice (Figure 3a). Additionally, numerous megakaryocytes were noted in DKO spleens (Figure 3a,b). Immunofluorescence (IF) staining for F4/80, a macrophage marker, showed decreased numbers of macrophages in the spleens of DKO mice compared to *Gsta4^−/−^* mice when housed in an SPF facility (Supplementary Figure S5A-C). We next stained for colon macrophages from *E. faecalis*- and sham-colonized mice and found that the number of macrophages in *E. faecalis*-colonized DKO mice was significantly decreased compared to sham-colonized DKO mice (Figure 3c,d), while there was no difference between *E. faecalis*-and sham-colonized *Gsta4^−/−^* mice (Figure 3c), *right panel* and 3E). Given that inactivation of GSTA4 increases intracellular ROS in CRC cells,^14^ and *Il10*^−/−^/*Gasta4*^−/−^ DKO mice have decreased the number of macrophages, we speculated that inactivation of Gsta4 may have induced ferroptosis in macrophages. We have determined the expression of Gpx4, a key regulator and marker of ferroptosis,^33^ in *E. faecalis*- and sham-colonized DKO mice. As anticipated, Gpx4 was decreased in colon biopsies from *E. faecalis*-colonized DKO mice compared to sham-colonized mice (Figure 3f,g). In contrast, Gpx4 was rarely observed in sham- or *E. faecalis*-colonized *Gsta4*^−/−^ mice.

### Inactivation of Gsta4 induces macrophage ferroptosis

To determine whether Gsta4 is associated with *E. faecalis*-induced macrophage ferroptosis, we inactivated Gsta4 in RAW264.7 cells. As shown in Supplementary Figure S6, we obtained a *Gsta4*-deficient cell line (RAW264.7^Δ*Gsta4*^ clone 2D6, henceforth termed 2D6) using guide RNA636 that targeted exon 6 of murine *Gsta4*. This cell line has a frameshift mutation caused by a deoxyadenosine insertion at position 109 in exon 6 of *Gsta4*.

**Figure 4.**
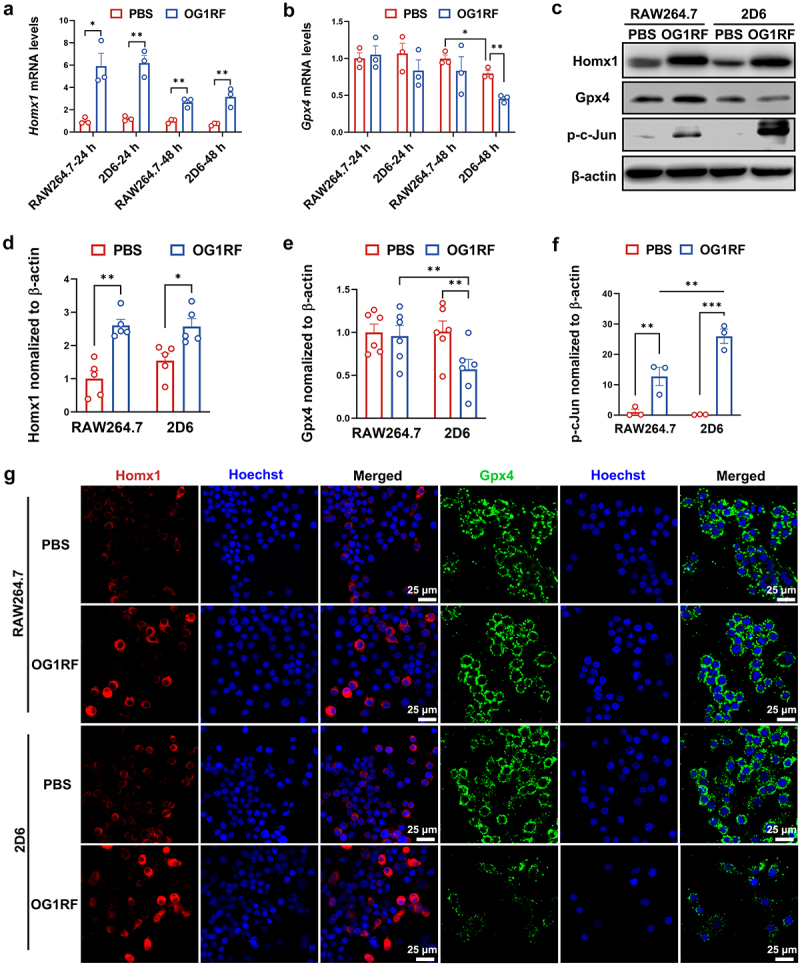
Inactivation of Gsta4 promotes *E. faecalis*-induced ferroptosis. (a) Quantitative real-time PCR shows significantly increased *Hmox1* expression in both RAW264.7 and *Gsta4*-deficient 2D6 cells infected with *E. faecalis* OG1RF compared to uninfected controls at 24 and 48 h. (b) qRT-PCR shows no change in *Gpx4* expression in both RAW264.7 and 2D6 cells 24 h following *E. faecalis* OG1RF infection, and decreased expression at 48 h, compared to PBS control. (c-f) Western blots confirm increased Hmox1 in both RAW264.7 and 2D6 cells following *E. faecalis* infection while decreased Gpx4 only in 2D6 cells following *E. faecalis* infection. Phosphorylated-c-jun is significantly increased in 2D6 cells following *E. faecalis* infection; all samples are at a 48-h time point post-infection. (g) Immunofluorescence staining shows no change in Hmox1 (*red*) in RAW264.7 and 2D6 cells and significantly decreased Gpx4 (*green*) in *E. faecalis*-infected 2D6 cells, but not RAW264.7 cells, compared to uninfected control. Nuclei (*blue*) are counterstained by Hoechst 33342. *p < 0.05; **p  < 0.01; ***p < 0.001; comparisons without *p* values are not statistically significant.

Ferroptosis is characterized by upregulation of *Hmox1*. Therefore, we determined *Hmox1* and *Gpx4* expression in *E. faecalis*-infected RAW264.7 and 2D6 cells. Quantitative RT-PCR showed increased expression of *Hmox1* in both cell lines at 24 and 48 h following *E. faecalis* infection compared to uninfected controls (Figure 4a). No decrease in *Gpx4* expression was noted in 2D6 cells compared to RAW264.7 cells at 24 h after the treatment (Figure 4b). In contrast, *Gpx4* expression was significantly decreased in 2D6 cells at 48 h after *E. faecalis* treatment compared to sham (Figure 4b). Western blots confirmed increased Hmox1 and decreased Gpx4 in *E. faecalis*-infected 2D6 cells compared to uninfected controls (Figure 4c-e). Furthermore, because AP-1 suppresses Gpx4 expression and thus aggravates ferroptosis,^34^ we determined the expression of phosphorylated c-Jun (p-c-Jun), a member of AP-1 transcription factor family, in *E. faecalis*-infected macrophages. As shown in Figure 4c,f, p-c-Jun increased in *E. faecalis*-infected RAW264.7 cells and further increased in *E. faecalis*-infected 2D6 cells compared to uninfected controls. Finally, IF staining confirmed increased Hmox1 in *E. faecalis*-infected RAW264.7 and 2D6 cells, and decreased Gpx4 in *E. faecalis*-infected 2D6 cells, but not RAW264.7 cells, compared to uninfected controls (Figure 4g). Taken together, these results indicate that extracellular superoxide-producing *E. faecalis* promotes ferroptosis in *Gsta4*-deficient macrophages via activating transcription factor c-Jun.

### Inactivation of Gsta4 increases oxidative stress in macrophages

**Figure 5.**
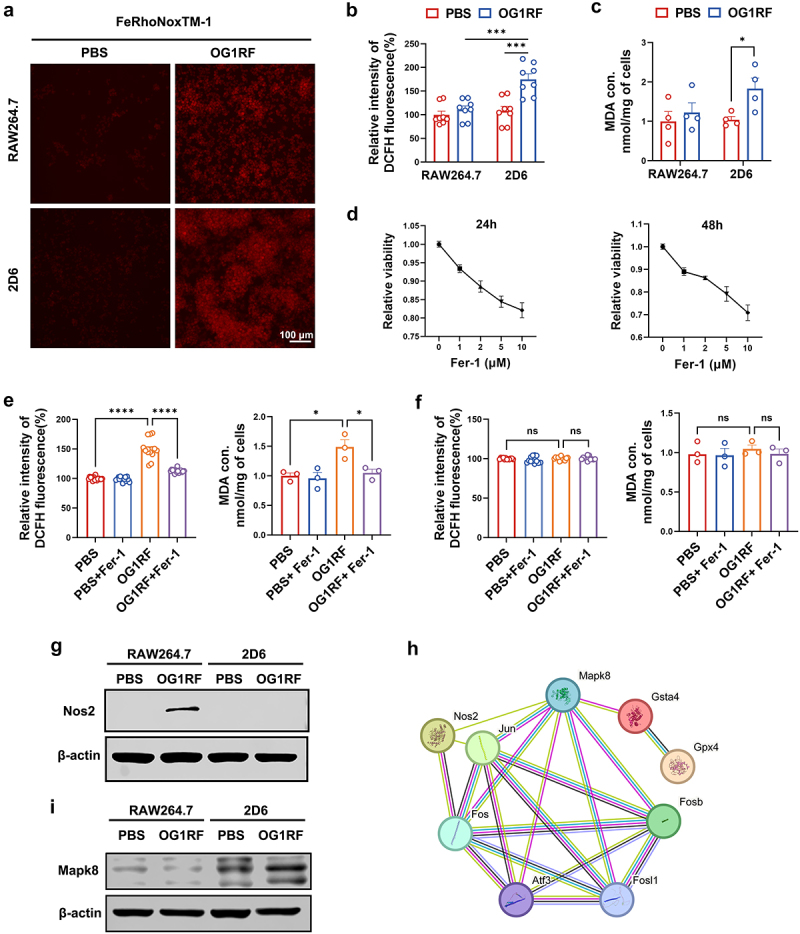
Inactivation of Gsta4 increases oxidative stress in macrophages. (a) Intracellular ferrous iron increases in both RAW264.7 and 2D6 cells 48 h following infection with *E. faecalis* OG1RF compared to uninfected PBS control. Increases in ferrous iron are noted in 2D6 cells compared to RAW264.7 cells after *E. faecalis* infection. (b and c) intracellular ROS and the lipid peroxidation product MDA significantly increase in *E. faecalis*-infected 2D6 cells but not RAW264.7 cells compared to uninfected control. (d) RAW264.7 cell viability following exposure to Fer-1 for 24 and 48 h; treatment with 1 µM Fer-1 for 24 h resulted in a > 90% survival rate and was selected for subsequent experiments. (e and f) *E. faecalis*-induced ROS and MDA are reduced by 1 µM Fer-1 at 24 h (e), while no changes in RAW264.7 cells (f). (g) Western blots show increased Nos2 in *E. faecalis*-infected RAW264.7 cells, but not 2D6 cells, compared to uninfected controls. (h) Predicted protein–protein interactions during ferroptosis using STRING database. (i) Western blots show increases in expression of Mapk8 in *Gsta4*-deficient 2D6 cells compared to RAW264.7 cells. *E. faecalis*-infection further upregulates Mapk8 expression. All data represent mean ± SEM from at least three independent experiments. *p < 0.05; ***p < 0.001; ****p < 0.0001; comparisons without *p* values are not statistically significant (ns).

We next examined intracellular ferrous iron and lipid peroxidation in *E. faecalis*-infected macrophages because ferroptosis is characterized by the accumulation of ferrous iron and lipid peroxidation products. Fluorescent staining showed that ferrous iron increased in *E. faecalis*-infected macrophages (Figure 5a). Analysis for intracellular ROS showed significant increases in *E. faecalis*-infected 2D6 cells compared to uninfected 2D6 cells, whereas no increase in ROS was observed in *E. faecalis*-infected RAW264.7 cells (Figure 5b). Likewise, the lipid peroxidation biomarker MDA was significantly increased in *E. faecalis*-infected 2D6 cells, but not RAW264.7 cells, compared to uninfected controls (Figure 5c).

To confirm *E. faecalis*-induced ferroptosis in *Gsta4*-deficient macrophages, RAW264.7 cells were treated with the ferroptosis inhibitor Fer-1, and >90% cells survived from 1 μM Fer-1 treatment (Figure 5d). Treatment with 1 μM Fer-1 reduced intracellular ROS and MDA in *E. faecalis*-infected 2D6 cells, but not RAW264.7 cells, compared to uninfected controls (Figure 5e,f). Because inducible nitric oxide synthase (Nos2) is a scavenger of reactive nitrogen species (RNS) and a key regulator of ferroptosis,^35^ we therefore investigated Nos2 expression in *Gsta4*-deficient macrophages. Consistent with previous reports,^4^ Western blots showed increased Nos2 expression in *E. faecalis*-infected RAW264.7 cells, indicating M1 polarization. In comparison, Nos2 expression was undetectable in *E. faecalis*-infected 2D6 cells (Figure 5g). These data indicate that inactivation of Gsta4 facilitates *E. faecalis*-induced intracellular ROS/RNS production, ferrous iron overloading, and lipid peroxidation while inhibiting Nos2 expression, leading to macrophage ferroptosis.

In addition, to determine the association of Gsta4 with ferroptosis, we analyzed the protein–protein interaction network of ferroptosis-associated proteins. STRING analysis showed that Gsta4 was closely connected with Gpx4, Nos2, and Mapk8 in the ferroptosis network (Figure 5h). Western blots also showed upregulation of Mapk8 in untreated 2D6 cells, which further increased after *E. faecalis* infection (Figure 5i), suggesting that inactivation of Gsta4 promotes ferroptosis by upregulating Mapk8.

**Figure 6.**
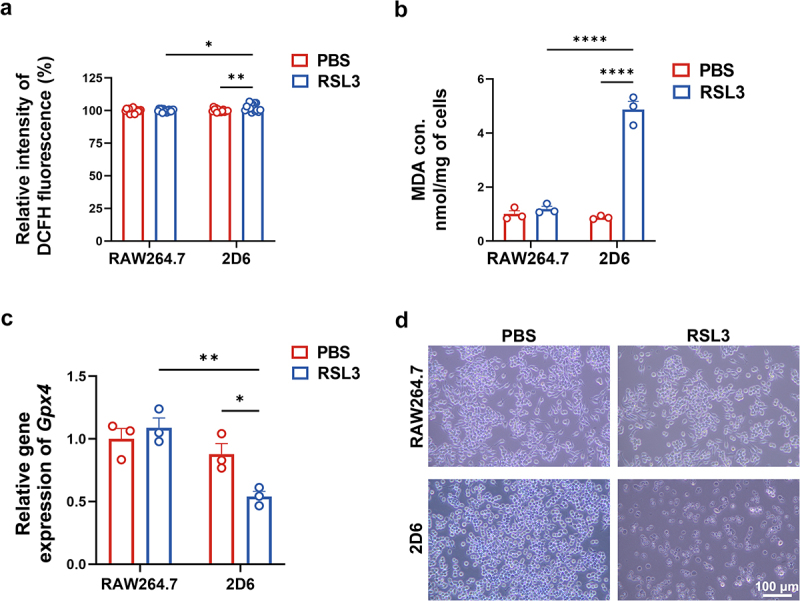
Inactivation of Gsta4 facilitates chemical-induced macrophage ferroptosis. (a) ROS is significantly increased in RSL3-treated 2D6 cells, but not RAW264.7 cells, compared to untreated controls. (b) MDA is also increased in RSL3-treated 2D6 cells, but not RAW264.7 cells, compared to untreated controls. (c) qRT-PCR shows reduced *Gpx4* expression in RSL3-treated 2D6 cells compared to untreated control or RSL3-treated RAW264.7 cells. (d) Representative photomicrographs for RAW264.7 and 2D6 cells treated with or without RSL3 for 20 h. Cell death is observed in RSL3-treated 2D6 cells. *p < 0.05; **p < 0.01; ****p < 0.0001; comparisons without *p* values are not statistically significant.

Furthermore, to investigate whether Gsta4 specifically blocks *E. faecalis*-induced ferroptosis, RAW264.7 and 2D6 cells were treated with the Gpx4 inhibitor RSL3. Similar to the effect of *E. faecalis* infection, RSL3 treatment significantly elevated ROS and MDA levels, reduced *Gpx4* expression, and triggered cell death, specifically, in 2D6 cells, but not in RAW264.7 cells (Figure 6a-d). These findings further underscore the protective role of Gsta4 in macrophages, not only in mitigating *E. faecalis*-induced ferroptosis but also in safeguarding against chemical-induced ferroptosis.

### GSTA4 expression is partially correlated with GPX4 expression in human CRC and IBD patients

**Figure 7.**
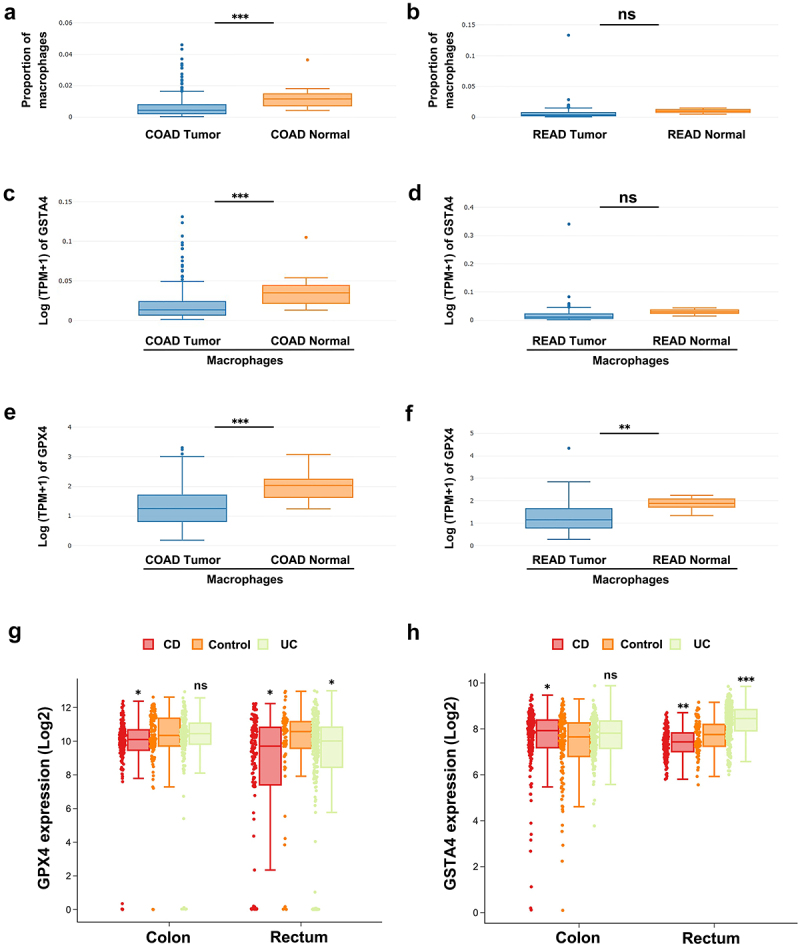
Expression of GSTA4 and GPX4 in human CRC and IBD patients. (a and b) TCGA analysis shows decreased macrophage proportion in human COAD (*n* = 290) compared to normal tissue (*n* = 41). No significant difference is noted in the proportion of macrophages in READ (*n* = 93) compared to normal tissue (*n* = 10). (c and d) Expression of GSTA4 in macrophages of human COAD, but not READ, is decreased compared to corresponding normal tissue. (e and f) Expression of GPX4 is decreased in macrophages of both human COAD and READ compared to normal tissue. (g and h) gene expression of GPX4 (g) and GSTA4 (h) in colon and rectal biopsies from IBD patients compared to healthy controls. CD, Crohn’s disease; UC, ulcerative colitis; control, healthy control. *p < 0.05; **p < 0.01; ***p < 0.001; ns, not significant.

To investigate the association of GSTA4 with macrophage ferroptosis in human CRC, we analyzed macrophage-specific expression of *GSTA4* and *GPX4* along with macrophage proportions in human colon adenocarcinomas (COAD) and rectal adenocarcinomas (READ) using TCGA database. TCGA analysis showed decreased proportions of macrophages in human COAD (*p* < 0.001), but not READ (*p* = 0.51), compared to the corresponding normal tissue (Figure 7a,b). Notably, macrophage-specific expression of *GSTA4* significantly decreased in COAD (*P* < 0.001), but not READ (*p* = 0.42), compared to normal tissue (Figure 7c,d). Macrophage-specific expression of *GPX4* decreased in both human COAD and READ compared to the corresponding normal tissues (*p* < 0.001 and 0.01 for COAD and READ, respectively; Figure 7e,f), suggesting that decreased expression of *GSTA4* may be associated with macrophage ferroptosis in human colorectal cancer.

Finally, analysis of gene expression in IBD patients revealed that *GPX4* expression decreased in both colon and rectum biopsies of Crohn’s disease (CD) patients as well as in the rectum of ulcerative colitis (UC) patients compared to healthy controls (Figure 7g). However, *GSTA4* expression varied across disease types and biopsy sections, with increasing in the colon of CD patients and rectum of UC patients, while decreasing in the rectum of CD patients compared to healthy controls (Figure 7h).

## Discussion

**Figure 8.**
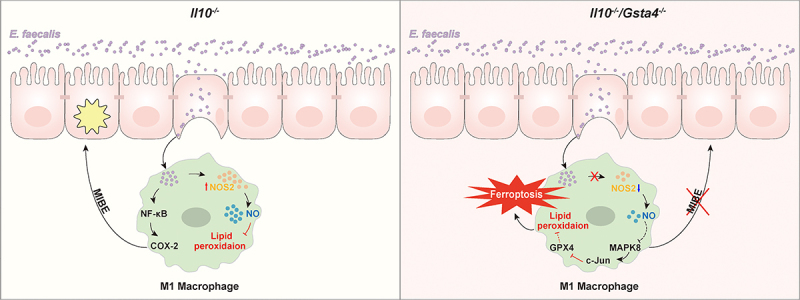
Inactivation of Gsta4 promotes macrophage ferroptosis and inhibits the microbiota-induced bystander effect (MIBE). When *Il10* is deficient, *E. faecalis* is able to polarize colon macrophages that express *Nos2* and produce inflammatory cytokines and mutagens leading to DNA damage, mutations, and neoplastic transformation in neighboring epithelial cells via a bystander effect. *Gsta4* is intact in these mice and their macrophages are tolerant to *E. faecalis*-induced oxidative damage. This permits the development of MIBE and CRC (*left*). In contrast, inactivation of *Gsta4* reduces the expression of *Gpx4* and *Nos2* while activates ferroptosis driver *Mapk8* leading to ferroptosis in macrophages following *E. faecalis* infection. The result is inhibition of MIBE and CRC (*right*).

Gut microbiota are important drivers for colitis and CRC. To date, no epidemiological data have shown the connection between specific pathobionts with human CRC, although several bacterial species are capable of inducing CRC in animal models, and feces from CRC patients can promote murine intestinal carcinogenesis.^2,36^ *E. faecalis* is a human commensal that induces CRC in *Il10^−/−^* mice through the MIBE.^3^ In *Il10*-deficient mice, *E. faecalis* polarizes colonic macrophages to an M1 phenotype that produces endogenous mutagens and inflammatory cytokines such as 4-HNE and TNFα, leading to mutations, chromosomal instability, and ultimately CRC.^3,4,37^ In *Il10*-deficient mice with functional *Gsta4* expression, macrophages are tolerant to *E. faecalis* infection-induced oxidative damage, which allows the occurrence of MIBE leading to CRC (Figure 8, *left*). In contrast, inactivation of Gsta4 inhibits *E. faecalis*-induced Nos2 expression, while it induces the Mapk8/c-Jun signaling pathway resulting in macrophage ferroptosis in *E. faecalis*-colonized *Il10*^−/−^ mice. This results in a loss of macrophages, inhibition of MIBE, and prevention of CRC (Figure 8, *right*). These findings suggest that Gsta4 is required for macrophage survival in the oxidative environment induced by *E. faecalis* infection, thereby promotes MIBE and CRC development.

Il10 is an important anti-inflammatory cytokine that inhibits macrophage polarization, although it can also act as a pro-inflammatory cytokine by stimulating CD8^+^ T cells.^38^ When Il10 is present, colonic macrophages are tolerant to commensal-induced activation. However, in the absence of Il10, colonic macrophages can be polarized by certain commensals, for example, *E. faecalis* and *E. coli*, leading to inflammation and CRC.^3,39^ Compared to *Il10*^−/−^ mice, *Gsta4^−/−^* mice (with intact *Il10*) did not develop colitis after 9-month colonization with *E. faecalis*, suggesting a primary role of M1-polarized macrophages in *E. faecalis*-induced CRC. Notably, 19.6% of *Il10^−/−^/Gsta4^−/−^* mice spontaneously developed colitis and, occasionally dysplasia, while housed in an SPF environment, supporting key roles for anti-inflammatory and antioxidative genes in protecting against CRC. Interestingly, in comparison to SPF mice, colitis was attenuated in both sham- and *E. faecalis*-colonized *Il10*^−/−^/*Gsta4^−/−^* mice. These unexpected findings led to a focus on colonic macrophages – the key effector cells for MIBE. We observed significantly decreased Gpx4 expression and numbers of macrophages in *E. faecalis*-colonized *Il10^−/−^/Gsta4^−/−^* mice, indicating ferroptosis in *Il10^−/−^/Gsta4^−/−^* mice. Interestingly, ferroptosis occurred only in DKO mice, not *Gsta4^−/−^* mice, when colonized with *E. faecalis*, further supporting Il10 deficiency-induced macrophage polarization as a precondition for MIBE.

Ferroptosis is an iron-dependent programmed cell death characterized by the accumulation of ROS, RNS, and lipid peroxidation products. *Gsta4*-deficient mice show increased 4-HNE and MDA levels in tissues. These mice are susceptible to bacterial infection and skin cancer.^11,40^ Unexpectedly, in the present study, we found no evidence for increased colitis and CRC in *E. faecalis*-colonized *Il10*^−/−^/*Gsta4^−/−^* mice. Notably, *Gsta4^−/−^* mice instead showed increased splenic siderophages. This may be due to the conversion of excessive ferrous iron to hemosiderin and enhanced oxidative stress.^41^ Previous studies have shown that bacterial infection causes the accumulation of ferrous iron and lipid peroxidation products in RAW264.7 cells.^42^ We also noted this in both RAW264.7 and *Gsta4*-deficient 2D6 cells after *E. faecalis* infection. 2D6 cells were loaded with greater amounts of ferrous iron than RAW264.7 cells. Hmox1 promotes ferroptosis by increasing lipid peroxidation and iron overloading.^43^ However, Hmox1 was upregulated in both RAW264.7 and 2D6 cells after *E. faecalis* infection, which may contribute to ferrous iron overloading and ROS production.^44^ Finally, *E. faecalis* infected RAW264.7 cells highly expressed Nos2 that reduced RNS production leading to inhibition of ferroptosis.^4,35^ Inactivation of Gsta4 reduced *E. faecalis*-induced Nos2 expression resulting in increased RNS and enhanced ferroptosis. How Gsta4 interacts with Nos2 in the regulation of ferroptosis is unknown and under investigation in our laboratory.

Ferroptosis in cancer cells is known to inhibit proliferation and tumor growth.^45^ However, studies focusing on the impact of macrophage ferroptosis on colitis and the initiation of CRC are lacking. In the present study, we show for the first time that Gsta4 is a critical regulator for macrophage ferroptosis. While our animal studies have shed light on the role of Gsta4 in macrophage ferroptosis and CRC initiation, translating these findings to human CRC has challenge. In line with animal studies, our analysis of human data from TCGA and IBD databases suggests a potential link between reduced GSTA4 expression and macrophage ferroptosis, especially in cases of human colorectal cancer. Recent studies show that inhibiting macrophage ferroptosis ameliorates chemical-induced colitis^46^ and that M1-polarized macrophages are susceptible to ferroptosis.^47,48^ Unlike these findings, we observed ferroptosis in *Gsta4*-deficient 2D6 cells, not RAW264.7 cells, following *E. faecalis* infection, suggesting an important role for Gsta4 in regulating ferroptosis. Consistent with our findings, others have shown that upregulation of Gsta4 mitigates ferroptosis in acute kidney injury.^49^ Taken together, these findings hold significant promise for potential therapeutic interventions in human CRC. First, given the overexpression of GSTA4 in human CRC, our studies suggest that utilizing small molecules to target GSTA4 could serve as a feasible approach for CRC prevention and treatment. Second, the observed reduction in colitis through the induction of macrophage ferroptosis underscores the importance of macrophages in the pathogenesis of colitis-associated CRC, providing a new avenue for the prevention and treatment of colitis-associated CRC by utilizing ferroptosis inducers to selectively target pathogenic macrophages.

This study has several limitations. First, we used a mouse strain with constitutively deleted *Gsta4* in all cells. The inability to conditionally inactive *Gsta4* may have led to systemic responses such as increased susceptibility to bacterial infection and skin cancer.^11^ Indeed, we observed pathophysiological changes in extraintestinal organs and cells such as increased splenic megakaryocytes and, occasionally, alopecia. In addition, we recently showed that inactivation of GSTA4 in human CRC cells inhibits proliferation and cancer growth.^14^ Whether *Gsta4*-deficiency promotes ferroptosis in colonic epithelial cells or other stromal cells besides macrophages, and thereby contributes to reduced colitis in *E. faecalis*-colonized *Il10*^−/−^/*Gsta4^−/−^* mice, remains unclear. Further research is warranted to fully elucidate the potential cancer preventive effect of Gsta4 deficiency through the use of organoid, patient-derived xenograft, and other CRC models. Second, because antibiotics may potentially reduce the degree of colitis,^50^ antibiotic use could have had an adverse effect on tumorigenesis as previously reported.^51^ As shown in previous studies,^3,7^ cancers fail to develop in control mice treated with antibiotics. Furthermore, we were unable to analyze the composition of gut microbiota for mice spontaneously developed colitis and CRC. This could help identify individual bacterial species responsible for colitis in DKO mice. This is a topic of further interest but was beyond the focus of this study.

Finally, 4-HNE is produced by *E. faecalis*-infected RAW264.7 cells and can induce apoptosis in these cells.^9,52^ As such, in addition to ferroptosis, RAW264.7 cells may potentially die via apoptosis after *E. faecalis* infection. Zou, et al. reported that *E. faecalis* at a low MOI can inhibit apoptosis in RAW264.7 cells.^53^ A recent study showed a role of Gsta4 in the regulation of oligodendrocyte apoptosis.^54^ However, our studies revealed that inactivation of GSTA4 in human CRC cells had no influence on chemotherapeutic agent-induced apoptosis.^14^ Whether the loss of Gsta4 promotes apoptosis in *E. faecalis*-infected macrophages remains uncertain, which is an ongoing project in our laboratory.

In conclusion, Gsta4 protects macrophages against *E. faecalis*-induced ferroptosis, which helps drive inflammation, MIBE, and the development of CRC. In contrast, inactivation of Gsta4 inhibits MIBE by promoting macrophage death through ferroptosis and thus blocks *E. faecalis*-induced colitis and CRC. These findings reinforce the key role of polarized macrophages in MIBE and provide novel insights into the mechanisms by which Gsta4 participates in the regulation of ferroptosis. These findings may lead to new strategies for CRC prevention and treatment.

## Supplementary Material

Supplemental Material

## Data Availability

All the data supporting our findings in the study are included in the paper.
