# Supplementary material for: Inactivation of glutathione S-transferase alpha 4 blocks Enterococcus faecalis-induced bystander effect by promoting macrophage ferroptosis
Source: Gut Microbes. 2025 Jan 16;17(1):2451090. doi: 10.1080/19490976.2025.2451090 (PMC11740687; doi:10.1080/19490976.2025.2451090)
Supplement: Supplemental Material [file KGMI_A_2451090_SM2832.zip › Supplementary material (2).docx]

**Supplementary Table S1. Guide RNA and PCR primers used in this study**

| gRNA and Primers | Sequence |
| --- | --- |
| *gRNA636* | AGAGGGAAGTCGGACAGTACAGG |
| *mGsta4-D-F2* | GTCTCTGTCCTGGTGGATAC |
| *mGsta4-D-F3* | GAACTCAGTGCCCCTGTACT |
| *mGsta4-D-R1* | GCCGGTCATGTGTTTAACTG |
| *Hmox1* |  |
| forward | CAAAGACCAGAGTCCCTCACA |
| reverse | GTCTGGGATGAGCTAGTGCTG |
| *Gpx4* |  |
| forward | CCGGCTACAACGTCAAGTTT |
| Reverse | ACGCAGCCGTTCTTATCAAT |

**Supplementary Table S2. Primary antibodies used in this study**

| Application/ Catalogue  Antigen Maker number Host Clonality Dilution |
| --- |
| *Western blotting*  Hmox1 Proteintech 10701-1-AP rabbit Poly 1:4,000  Gpx4 Proteintech 67763-1-Ig mouse Mono 1:4,000  p-c-Jun Santa Cruz Biotechnology sc-822 mouse Mono 1:400  Nos2 Santa Cruz Biotechnology sc-7271 mouse Mono 1:500  Mapk8 MCE HY-P80831 rabbit Mono 1:600  β-Actin CST 3700 mouse Mono 1:1,000  *IF and IHC staining*  F4/80 CST 30325 rabbit Mono 1:300  Hmox1 Proteintech 10701-1-AP rabbit Poly 1:500  Gpx4 Proteintech 67763-1-Ig mouse Mono 1:500  Pcna CST 2586 mouse Mono 1:1,000  TNFα Sangon D164309 rabbit Poly 1:100  Il6 Affinity DF6087 rabbit Poly 1:2,000 |

**Supplementary Figure Legends**

**Supplementary Figure S1.** **No signs of spontaneous colitis are observed in *Gsta4^-/-^* mice housed in a SPF environment.** H&E staining shows normal histopathology without inflammation in colons and rectum.

**Supplementary Figure S2. *E. faecalis* fails to induce colitis in *Il10*^-/-^/*Gsta4^-/-^* mice. (A)** Representative photomicrographs of H&E staining for biopsies from mid-colon and proximal colon. (B) Inflammation scores for biopsies of mid- and proximal colons from *E. faecalis* OG1RFSS- (n = 8) and sham-colonized (n = 6) mice.

**Supplementary Figure S3. *E. faecalis* colonization does not induce colitis or CRC in *Gsta4^-/-^* mice. (A)** H&E staining shows normal histopathology in colorectal biopsies from *E. faecalis* OG1RFSS- and sham-colonized *Gsta4^-/-^* mice. **(B)** Inflammation scores for colorectal biopsies. n = 6 and n = 8 for sham and OG1RFSS group, respectively. **(C and D)** Representative photomicrographs of immunohistochemical staining for inflammatory cytokines TNFα and Il6 in colorectal biopsies from *E. faecalis* OG1RFSS- and sham-colonized *Gsta4^-/-^* mice. **(E and F)** Immunohistochemical staining scores for colorectal biopsies from *E. faecalis* OG1RFSS- and sham-colonized mice. **(G)** AB-PAS staining shows mucosal integrity in colorectal biopsies from both OG1RFSS- and sham-colonized mice. ns, not significant.

**Supplementary Figure S4. Colonization of *Il10*^-/-^/*Gsta4^-/-^* and *Gsta4^-/-^* mice with *E. faecalis* does not increase proliferation. (A)** Immunofluorescent staining for proliferating cell nuclear antigen (Pcna) in the rectal biopsies from *E. faecalis* OG1RFSS- and sham-colonized *Il10*^-/-^/*Gsta4^-/-^* and *Gsta4^-/-^* mice. **(B)** Fluorescence intensity for rectal biopsies from OG1RFSS- and sham-colonized mice. **(C)** Pcna staining for biopsies of distal colons from *E. faecalis* OG1RFSS- and sham-colonized *Il10*^-/-^/*Gsta4^-/-^* and *Gsta4^-/-^* mice. **(D)** Similarly, no differences are observed between OG1RFSS- and sham-colonized mice. ns, not significant.

**Supplementary Figure S5.** **Inactivation of Gsta4 is associated with macrophage depletion.** IF staining shows decreased F4/80-positive macrophages in spleens of *Il10*^-/-^/*Gsta4^-/-^* mice **(B)** compared to *Gsta4^-/-^* mice **(A)** when housed in an SPF facility. **(C)** Relative density of macrophages in spleens from these two strains. ***P* < 0.01.

**Supplementary Figure S6. Inactivation of Gsta4 in RAW264.7 cells. (A)** The schematic diagram for the partial sequence of murine *Gsta4* gene showing the positions of gRNA and primers. **(B)** PCR products of *Gsta4* amplified by primer set mGsta4-D-F3 and mGsta4-D-R1 and separated on 6% agarose gel. **(C** and **D)** Sanger sequencing demonstrates a deoxyadenosine insertion at position 109 in exon 6 of *Gsta4* gene. (**E**) Amino acid sequence alignment shows frame shift mutation (*red*) in Gsta4 in 2D6 cells.
