# Supplementary figures and images for: Inactivation of glutathione S-transferase alpha 4 blocks Enterococcus faecalis-induced bystander effect by promoting macrophage ferroptosis
Source: Gut Microbes. 2025 Jan 16;17(1):2451090. doi: 10.1080/19490976.2025.2451090 (PMC11740687; doi:10.1080/19490976.2025.2451090)

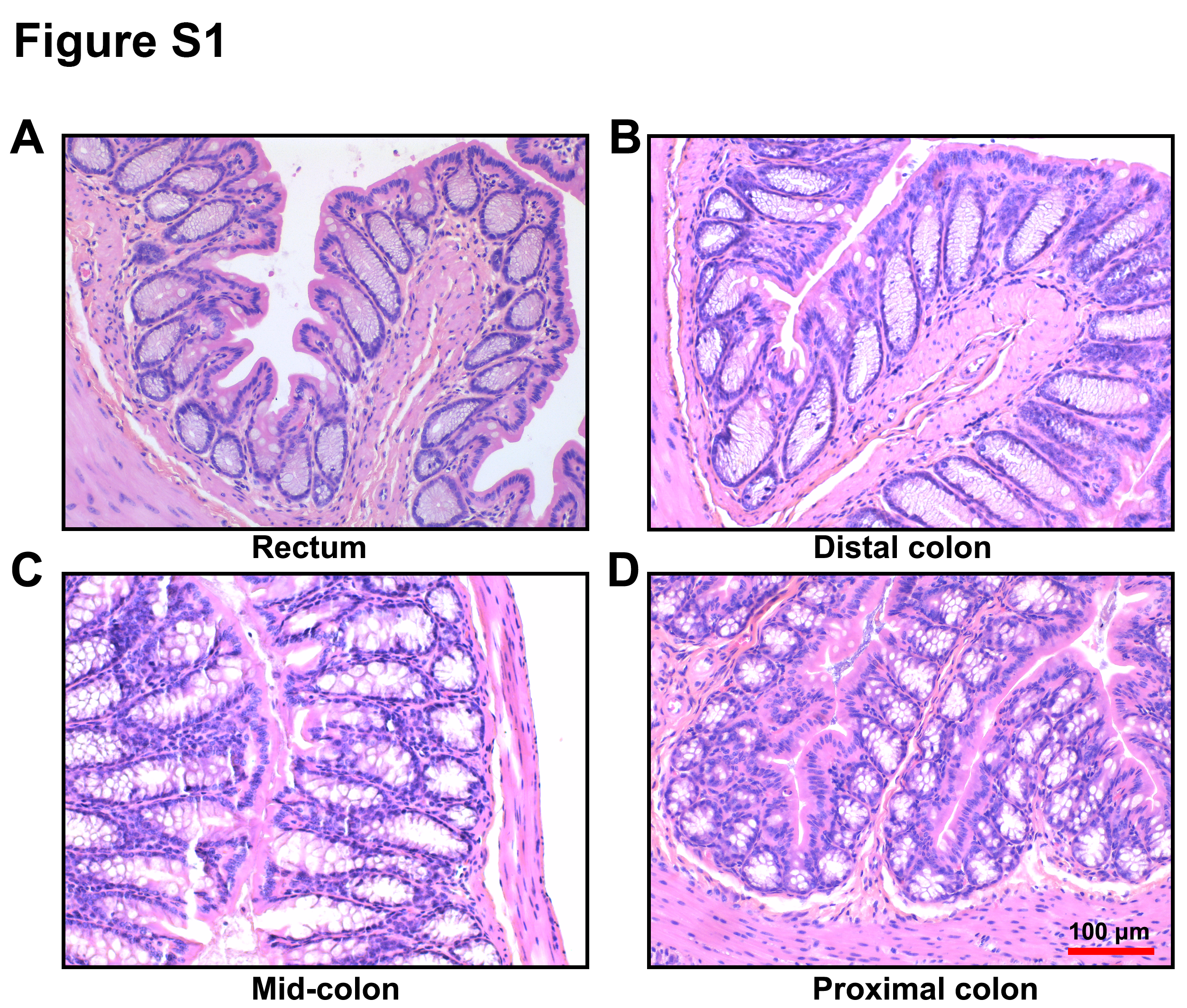

Supplement: Supplemental Material [file KGMI_A_2451090_SM2832.zip › figureS1.tif]

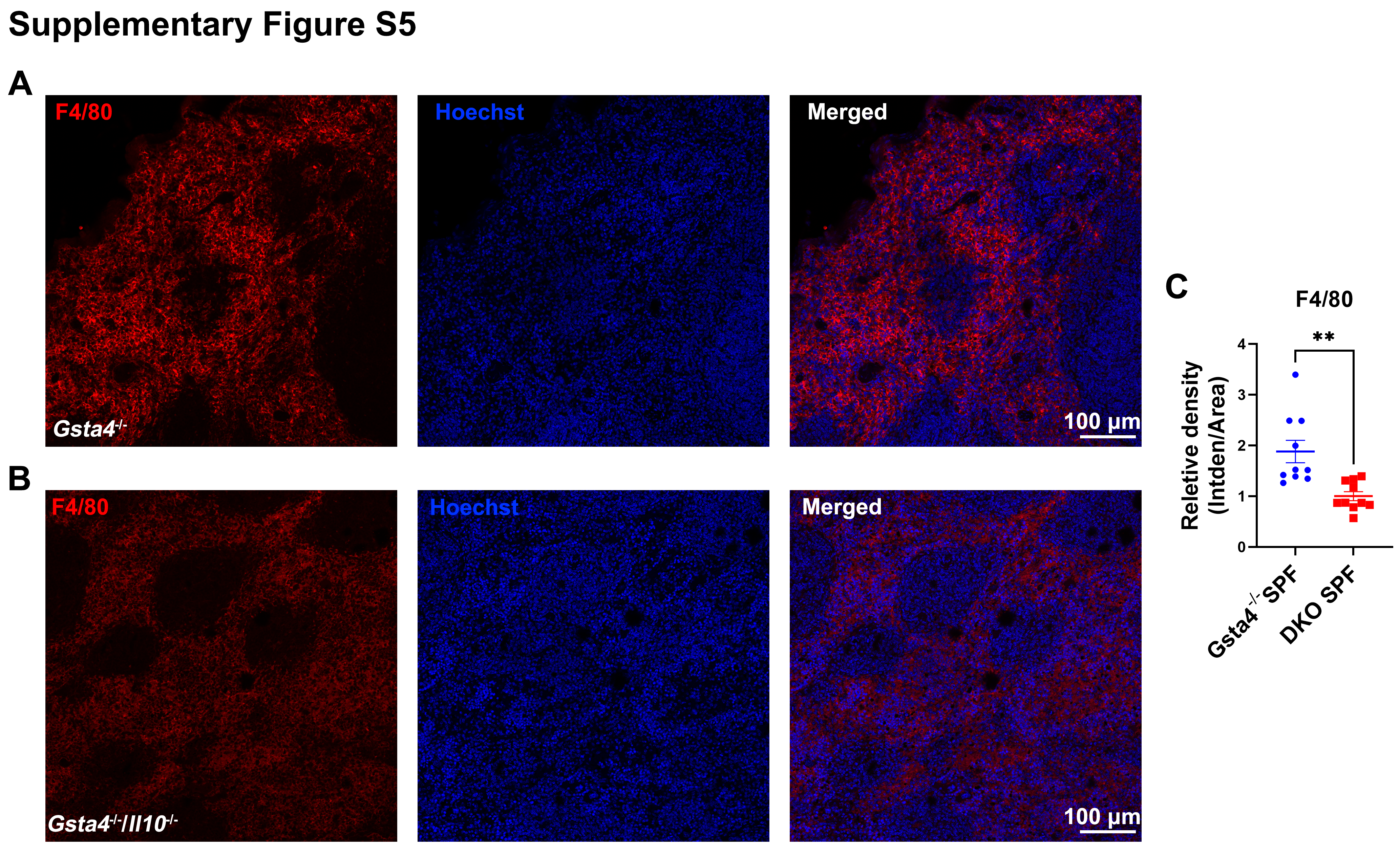

Supplement: Supplemental Material [file KGMI_A_2451090_SM2832.zip › figureS5.tif]

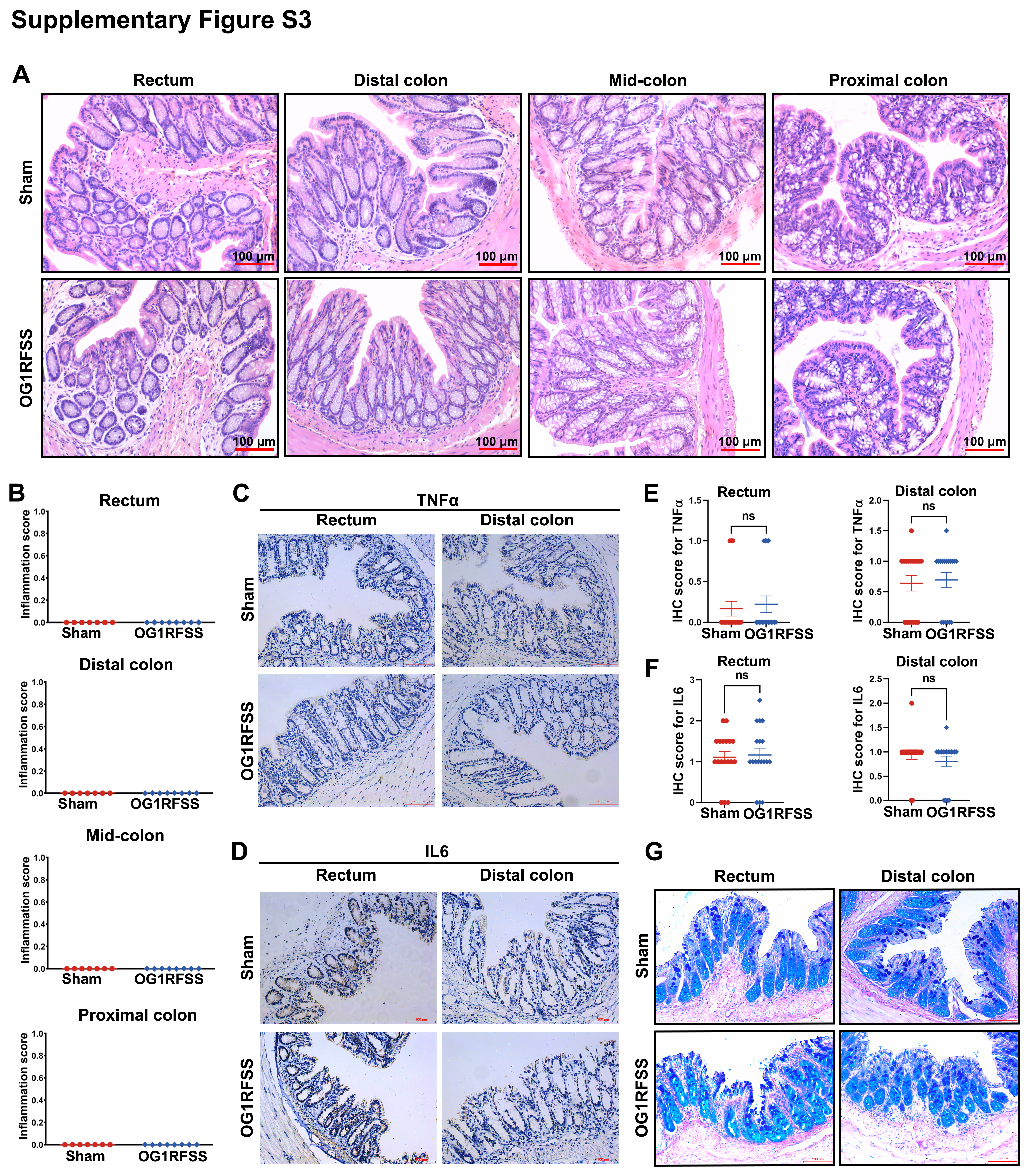

Supplement: Supplemental Material [file KGMI_A_2451090_SM2832.zip › FigureS3.tif]

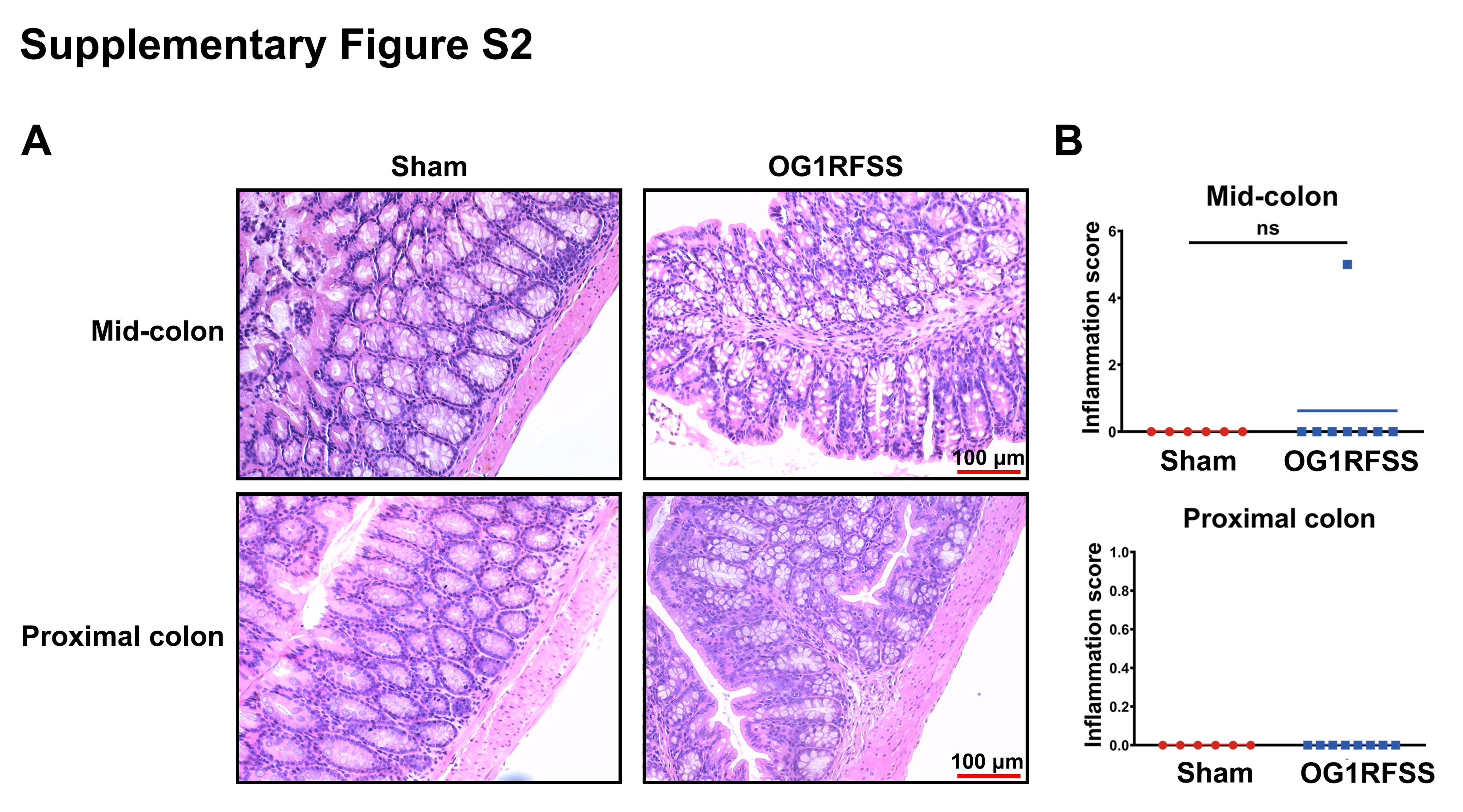

Supplement: Supplemental Material [file KGMI_A_2451090_SM2832.zip › FigureS2.tif]

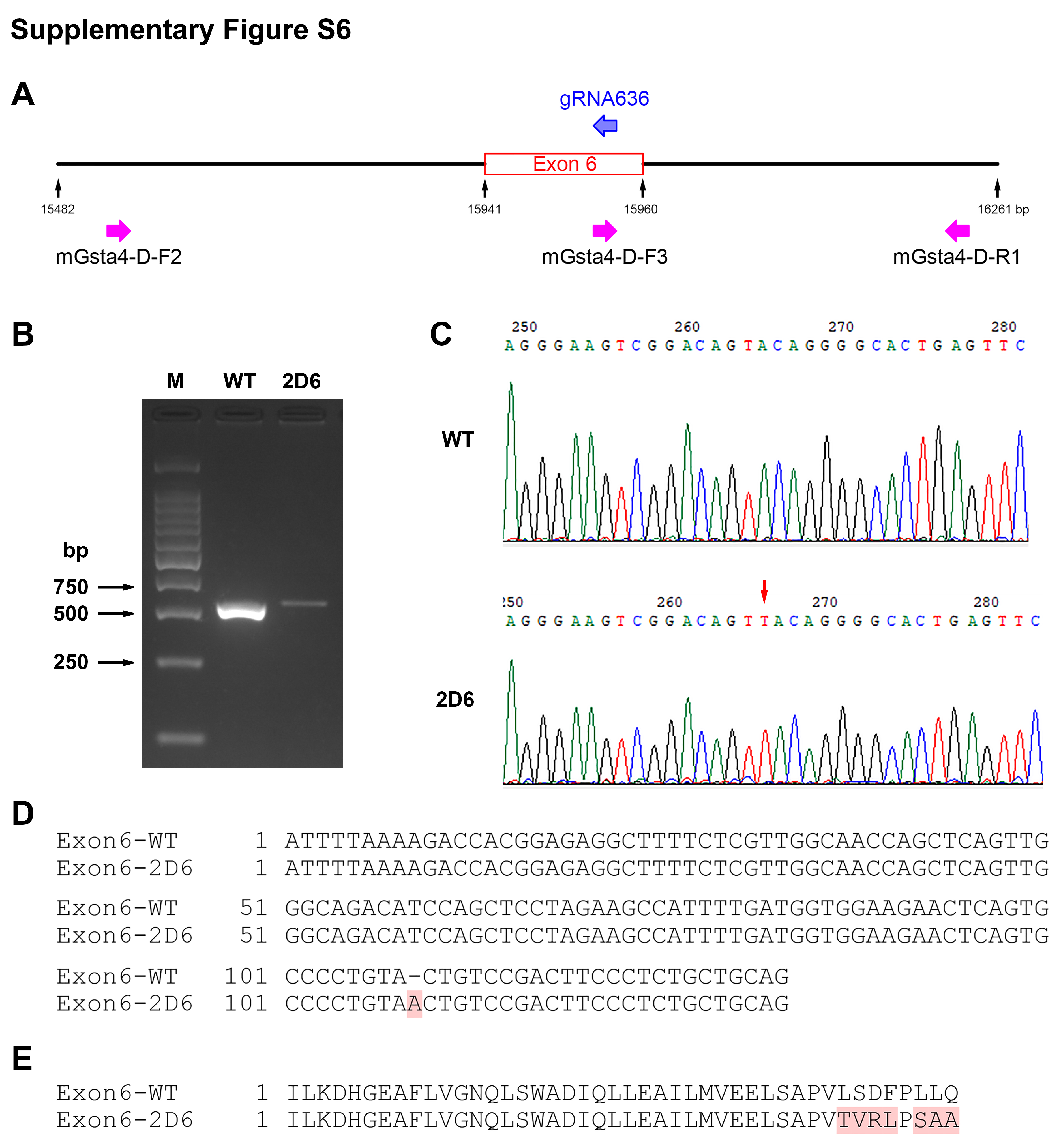

Supplement: Supplemental Material [file KGMI_A_2451090_SM2832.zip › FigureS6.tif]

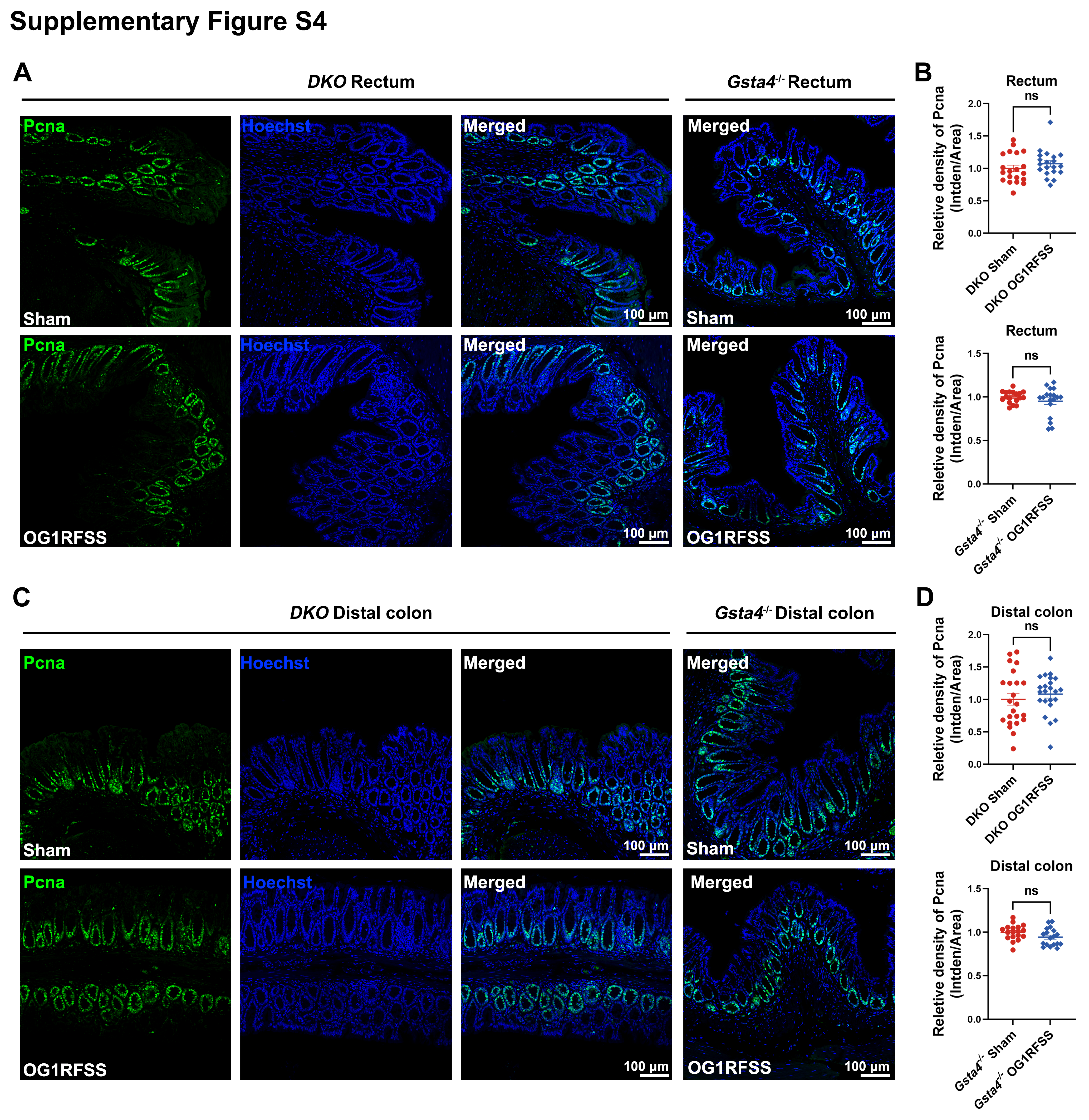

Supplement: Supplemental Material [file KGMI_A_2451090_SM2832.zip › FigureS4.tif]
